# THE DERMATOSES OF PREGNANCY

**DOI:** 10.4103/0019-5154.43203

**Published:** 2008

**Authors:** Silonie Sachdeva

**Affiliations:** *From Jalandhar, Punjab, India*

**Keywords:** *Dermatoses*, *pregnancy*, *skin*

## Abstract

The skin changes in pregnancy can be either physiological (hormonal), changes in pre-existing skin diseases or development of new pregnancy specific dermatoses. Pregnancy-specific skin dermatoses include an ill-defined heterogeneous group of pruritic skin eruptions which are seen only in pregnancy. These include atopic eruption of pregnancy, polymorphic eruption of pregnancy, pemphigoid gestationis and intrahepatic cholestasis of pregnancy. Atopic eruption of pregnancy is the most common of these disorders. Most skin eruptions resolve postpartum and require only symptomatic treatment. Antepartum surveillance is recommended for patients with pemphigoid gestationis and intrahepatic cholestasis of pregnancy as they carry fetal risk. This article deals with the classification, clinical features and treatment of the specific dermatoses of pregnancy.

## Introduction

Pregnancy is characterized by many physiological skin changes as striae gravidarum, melasma accompanied by hair, nail and vascular changes which are due to hormonal effects. Along with this, the pre-existing skin conditions may either improve or exacerbate in pregnancy due to immunological changes in pregnancy. As cell mediated immunity is depressed during normal pregnancy, this accounts for increased severity and frequency of skin infections as candidiasis. There are however few inflammatory skin dermatoses which are specific to pregnancy and are seen only in pregnancy. Though most of these skin dermatoses are benign and resolve in postpartum period, a few can risk fetal life and require antenatal surveillance.

## Classification

The first classification of dermatoses of pregnancy was proposed by Holmes and Black^1^ in 1983 and included four skin conditions: 1. Pemphigoid gestationis (PG, syn. herpes gestationis) 2. Polymorphic eruption of pregnancy (PEP) (syn., pruritic urticarial papules and plaques of pregnancy [PUPPP]) 3. Prurigo of pregnancy (PP) and 4. Pruritic folliculitis of pregnancy (PF). The second, proposed by Shornick^2^ in 1998, included intrahepatic cholestasis of pregnancy (ICP) in addition to PG, PEP and PP. The most recent rationalized classification has been proposed by Ambros-Rudolph *et al*^3^ in 2006 after their retrospective two-center study on 505 pregnant patients. They introduced a new entity “Atopic eruption of pregnancy (AEP)” as AEP was observed to be the most common pruritic skin condition in pregnancy in their study. It was noted in almost 50% of patients affected with pregnancy specific dermatosis. They included three conditions- eczema in pregnancy (EP), prurigo of pregnancy and pruritic folliculitis of pregnancy under AEP due to their overlapping features. Therefore they presented four main conditions: 1. Atopic Eruption of Pregnancy, 2. Polymorphic eruption of Pregnancy, 3. Pemphigoid Gestationis and 4. Intrahepatic Cholestasis of Pregnancy under dermatoses of pregnancy. While AEP starts significantly earlier, PEP, PG, and ICP present in late pregnancy.

### 1. Atopic eruption of pregnancy (AEP) (Syn. early - onset prurigo of pregnancy)

#### Eczema in pregnancy:

Vaughan-Jones *et al*,^4^ in a prospective study on pruritic skin diseases in pregnancy in 1999 reported a high prevalence of atopic eczema in pregnancy for first time. This finding was not reflected in the previous classifications^1^–^2^ which had concluded polymorphic eruption of pregnancy as the most common dermatosis in pregnancy. The reason for increased incidence of atopic eczema in pregnancy was cited to be due to immunological changes in pregnancy. Pregnancy is characterized by a lack of strong maternal cell-mediated immune function and T-helper 1 (Th1) cytokine production (eg, IL-12, interferon gamma) and a dominant humoral immune response and T-helper 2 (Th2) cytokine production (e.g., IL-4, IL-10) to prevent fetal rejection.^5^ The Th2 shift associated with pregnancy may favor the exacerbation of atopic dermatitis. Ambros-Rudolph *et al*,^3^ confirmed these findings in a retrospective two-center study on 505 pregnant patients. In their study it was revealed that 80% of the affected patients experienced first episode of atopic eczema during pregnancy. These patients had an atopic background with raised total serum IgE levels. The eruption was seen more commonly in primigravida with single gestation pregnancy and skin lesions started during early pregnancy in first and second trimester. The skin eruption affected all parts of the body, including face, palms, and soles.

#### Prurigo of pregnancy:

Prurigo of pregnancy has been reported to occur in approximately one in 300 -pregnancies.^6^ It is characterized by pruritic, often excoriated papules and nodules on the extensor surfaces of the legs and upper arms. The abdomen can also be involved. The time of onset is variable and it has been reported to occur in all trimesters. The etiology and pathogenesis is not known, although there is sometimes a history of atopy.^4^ There are no recognized adverse effects for the mother or fetus.

#### Pruritic folliculitis:

This rare dermatosis occurs in the second and third trimester of pregnancy and affects an estimated one in 3,000 pregnancies.^7^ Contrary to its name, pruritus is not a major feature and it may be mistaken for acne or microbial folliculitis.^8^,^9^ It is characterized by an acneiform eruption consisting of multiple, pruritic, 2- to 4-mm, follicular papules or pustules typically on the shoulders, -upper back, arms, chest, and abdomen. The diagnosis is made clinically after excluding other, more common rashes. The skin lesions usually resolve spontaneously one to two months following delivery. Small case series have failed to implicate immunologic dysfunction or elevated androgen levels.^10^–^12^ On histopathology, an acute sterile folliculitis is evident and direct immunofluorescence stains are negative. The disorder is not associated with any maternal or fetal morbidity, although one small series of patients showed a reduction in fetal birth weight.^12^

### 2. Polymorphic eruption of pregnancy (Syn. late - onset prurigo of pregnancy)

Polymorphic eruption of pregnancy also known as pruritic urticarial papules and plaques of pregnancy (PUPPP), occurs with an incidence of one in 160 pregnancies^4^ and is the second most common skin dermatosis in pregnancy after atopic eczema. It is associated with multiple gestation and increased maternal weight gain. The exact etiology is not known. It has been proposed that stretching of the skin damages the connective tissue causing subsequent conversion of nonantigenic		ecules to antigenic ones, leading to skin eruption.^13^–^14^ PUPPP usually occurs in primigravidas in the third trimester and recurrence in subsequent pregnancies is unusual. The eruption may first appear in postpartum period. PUPPP has a marked pruritic component and the onset of pruritis coincides with the skin lesions which are seen as polymorphous, erythematous, nonfollicular papules, plaques, and sometimes vesicles. The eruption begins over the abdomen, commonly involving striae gravidarum with sparing of the periumbilical region. It may spread to the breasts, upper thighs, and arms. The face, palms, soles, and mucosal surfaces are usually spared. Histopathologic findings are nonspecific and the immunoflourosecence studies are negative. The lesions resolve near term or in the early postpartum period. The maternal and fetal prognosis is excellent.^14^,^15^

### 3. Pemphigoid gestationis (herpes gestationis)

Pemphigoid gestationis is a rare autoimmune disorder occurring in approximately one in 50,000 pregnancies, and begins in the second or third trimester. The condition has been linked to the presence of HLA-DR3 and HLA-DR4 and has a rare association with molar pregnancies and choriocarcinoma has been reported.^16^ It has been suggested that the disease could be triggered by a placental antigen that causes cross-reaction with skin antigens. This explains the onset of the disease in the periumbilical region. The skin lesions are pruritic, urticarial and vesiculobullous. Histologically, it is characterized by subepidermal vesicle formation, and immunopathologically by deposition of complement 3 along the basement membrane zone. These features are shared by another skin disease bullous pemphigoid which suggests that herpes gestationis may be a related entity.^17^ The condition may resolve late in pregnancy, but classically flares up again at delivery. Fetal risk has not been substantiated, although immunoglobulin G autoantibodies cross the placenta, and 5 to 10 percent of newborns have urticarial, vesicular or bullous lesions.^18^ Mild placental failure has been associated with premature deliveries and newborns that are small for gestational age. Therefore, antenatal surveillance is advised. Affected patients may have nongestational recurrences triggered by oral contraceptives and menstrual cycles.^6^

### 4. Pruritus gravidarum (intrahepatic cholestasis of pregnancy) (ICP)

The two terms term pruritus gravidarum and intrahepatic cholestasis of pregnancy (ICP) have been used interchangeably in the literature.^7^ While pruritus gravidarum is classically associated with itching, without any skin lesions and occurs in the first trimester, ICP (also called obstetric cholestasis) is seen in third trimester and is characterized by pruritus with or without jaundice, absence of primary skin lesions, and with laboratory markers of cholestasis.^19^ The skin lesions are usually secondary linear excoriations and excoriated papules, which are caused by scratching and are localized on the extensor surfaces of the limbs, abdomen and back. The severity of skin lesions correlates with the duration of pruritus.^3^ The etiology of ICP remains controversial. A family history of the condition is common, and there is an association with the presence of human leukocyte antigen-A31 (HLA-A31) and HLA-B8. The condition tends to recur in subsequent pregnancies.^7^,^19^ Patients may have a family history of cholelithiasis and a carry higher risk of gallstones.^20^,^21^ Laboratory markers include elevated serum bile acid levels (4.08 mcg per mL [10 μmol per L] or more) and alkaline phosphatase levels with or without elevated bilirubin levels. The condition is associated with a higher risk of premature delivery, meconium-stained amniotic fluid, and intrauterine demise due to anoxia caused by decreased fetal elimination of toxic bile acids.^22^ A statistically significant increase in adverse fetal outcomes has been reported in patients with bile acid levels of 16.34 mcg per mL (40 μmol per L) or more.^20^ Cholestasis and jaundice in patients with severe or prolonged intrahepatic cholestasis of pregnancy may cause vitamin K deficiency and coagulopathy.^19^ Therefore, early diagnosis, prompt treatment, and close obstetric surveillance are mandatory in cases of ICP.

## Treatment

Atopic eruption of pregnancy and polymorphic eruption of pregnancy are benign skin conditions and do not carry any fetal risk.^3^,^6^ They usually resolve in early post partum period. During pregnancy they respond well to topical emollients and moderately potent steroids in combination with oral antihistamines. Systemic steroids may be used for severe cases of PUPPP.^14^,^15^ Pruritic follicullitis responds well to topical benzoyl peroxide^8^,^9^ and also has been treated with narrowband (TL-01) ultraviolet B phototherapy.^23^ Patients with pemphigoid gestationis and intrahepatic cholestasis of pregnancy carry fetal risk and require specific treatment. The mild pruritus can be treated with oral antihistamines but patients with more severe cases of PG require systemic corticosteroids.^16^,^19^ PG has also been treated with high-dose intravenous immunoglobulins.^24^ For ICH, ursodeoxycholic acid is considered to be the drug of choice, as it is the only therapy that has been shown to decrease both maternal pruritus and fetal mortality.^25^,^26^
